# Notes and News

**Published:** 1896-03-14

**Authors:** 


### Notes and News.

(Collected and Communicated.)

### HOSPITAL MEETINGS.

The Right Hon. Sir Algernon West, K.C.B., will preside at the Anniversary Festival of the Reedham Orphanage, Purley, Surrey, to be held at the Hotel Metropole on Friday, March 27th.

The Annual Meeting of Governors of the Hospital and Homes for Incurable Children, 2, Maida Vale, W., will be held on Saturday, March 14th, at 3 p.m.

The Annual Meeting of the National Dental Hospital and College, Great Portland Street, W., will be held at the Hospital on Wednesday, March 25th, at 5 o'clock.

The Annual General Meeting of the Royal Albert Orphan Asylum, Bagshot, will be held at the offices, 62, King William Street, E.C., on Wednesday, March 18th, at 2 p.m.

The Anniversary Festival of the British Orphan Asylum, Slough, will be held at the Whitehall Rooms on April 22nd, under the presidency of Lord Rothschild.

The Annual General Meeting of the University College Hospital, Gower Street, W.C., will be held in the Board-room on Friday, March 20th, at 4 p.m.

The 83rd Anniversary Festival in aid of the London Orphan Asylum, Watford, will be held at the Grand Hotel on Thursday, April 30th, under the presidency of Mr. Edward Holroyd Bousfield.

The Royal Hospital for Incurables, West Hill, Putney Heath, will hold its annual dinner on Tuesday, May 19th, at the Hotel Metropole, under the chairmanship of Mr. H. J. Allcroft, the treasurer.

It has been decided to establish a cottage hospital in connection with the dispensary at Malton. The proposal met with much opposition, most of the medical men of the district disapproving of the measure.

A festival dinner in aid of the funds of the Royal National Hospital for Consumption, Ventnor, will be held at the Whitehall Rooms, Hotel Metropole, on Wednesday, April 29th, under the presidency of the Attorney-General (Sir Richard E. Webster).

A dinner has been given at Birmingham to Sir Willoughby Wade in honour of his recent knighthood. Sir Willoughby was presented with an illuminated address, and the speeches on the occasion were full of recognition of his excellent work.

Mr. Herbert Bluett has been appointed secretary of the City of London and East London Dispensary, in place of his father, Mr. F. Bluett, who has resigned the post. Mr. F. Bluett has been unanimously elected a member of the committee of management.

Dr. Cesare Lombroso has been made director of the University Clinic for Mental Diseases at Turin. He has also been transferred from the Professorship of Legal Medicine to that of Psychiatry in the same University.

The Pennsylvania Hospital is about to be enlarged through the generosity of an anonymous donor. The extension is to consist of a three-storey building one hundred and thirty-five feet in length and fifty in breadth. It is to be devoted to the purpose of receiving ward and general clinics.

The St. Petersburg School of Medicine for Women is fast being established on a firm basis. The endowment fund now amounts to £70,000. The Government and the city have both undertaken to provide large annual grants. The course of instruction has already commenced, though the school does not yet possess its own buildings.

It is hoped that a maternity hospital will soon be provided at Dundee. The Cobb trustees have granted £5,000 for the purpose, and a further like amount remains to be collected to secure the necessary £10,000. The committee which has been formed for the purpose have selected Logie House for the new institution. Attached to the house is a large garden. A considerable amount will have to be spent in adapting the house for use as a hospital.

Mr. Henry Tate, of Streatham, who was the original donor of the Liverpool Hahnemann Hospital, has now presented that institution with the sum of £5,000, to be invested for the purpose of the maintenance of the charity. Mr. J. Temple, speaking at the annual meeting of the institution, said he felt that proof of the appreciation in which Mr. Tate's generosity was held could not be better shown than by following his example by adding to the endowment fund. For this purpose he promised £500, and to start every £1,000, after the first, with £50. The institution has been fortunate enough in having found another friend who intends presenting to it a convalescent home for the admission of children and young women.

Mr. Conrad W. Thies, secretary of the Royal Free Hospital, expatiated at the Denison Club on the 4th inst. on the lot of a hospital secretary. Being the chief executive officer, in addition to a multiplicity of correspondence and the necessary statistics and accounts, the hospital secretary has to acquire knowledge of building operations and sanitation, the quality of goods of various kinds, and to display a readiness of resource in attracting and fixing public attention upon his institution so as to secure the money necessary for its maintenance. Mr. Thies was of opinion that multifarious and anxious as were the duties, the compensations attaching to the office exceeded them in interest, and well repaid the best efforts of the best men holding this important position.

